# Supplementary material for: The Role of Palmitoleic Acid in Regulating Hepatic Gluconeogenesis through SIRT3 in Obese Mice
Source: Nutrients. 2022 Apr 1;14(7):1482. doi: 10.3390/nu14071482 (PMC9003329; doi:10.3390/nu14071482)
Supplement: Supplementary file 1 [file nutrients-14-01482-s001.zip › nutrients-1628976-supplementary.pdf]

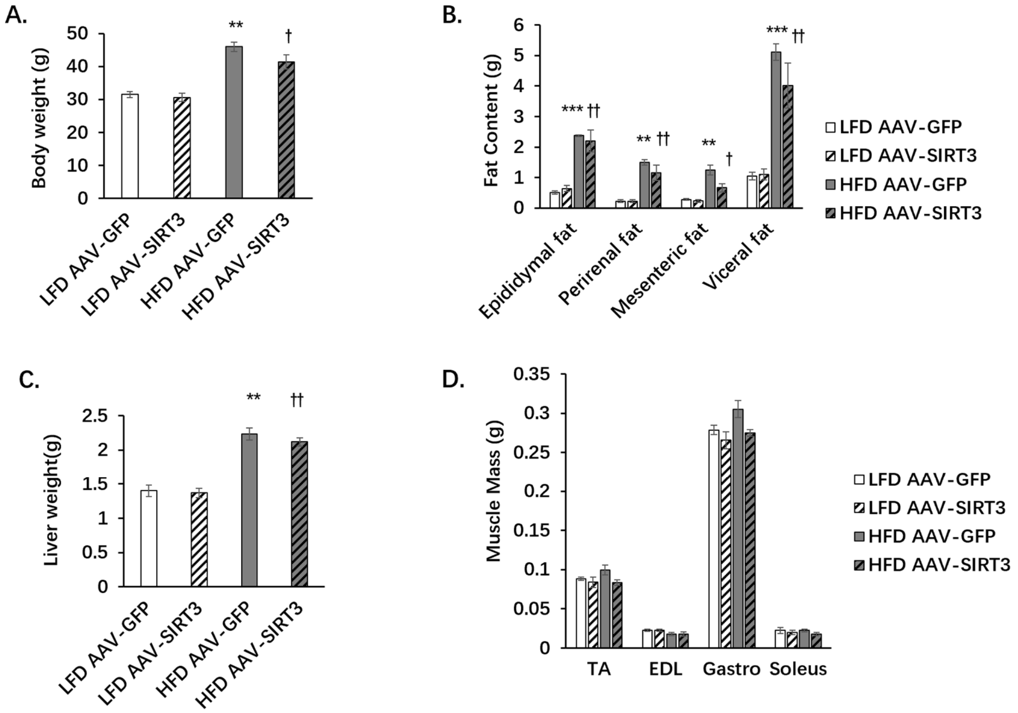

Figure S1. Liver specific SIRT3 overexpression does not change body weight and tissue weight in mice.5-6 weeks of C57BL/6N male mice were injected AAV-GFP or AAV-SIRT3 via tail vein and fed with LFD and HFD for 12 weeks, respectively. At the 14th weeks of feeding, mice were sacrifized and liver, fat and muscle tissue were collected from the mice. A. Body weight of mice. B. Fat content. Viceral fat includes epididymal fat, perirenal fat, and intestinal fat. C. Liver weight. D. Muscle weight. TA; anterior tibialis muscle; EDL; extensor digitorum longus; Gastro; gastrocnemius muscle.The data are mean ± s.e. (error bars). n=11-12 mice per group. *, $p<0.05$, **, $p<0.01$, ***, $p<0.001$, LFD AAV-GFP vs HFD AAV-GFP; †, $p<0.05$, ††, $p<0.01$, LFD AAV-SIRT3 vs HFD AAV-SIRT3.

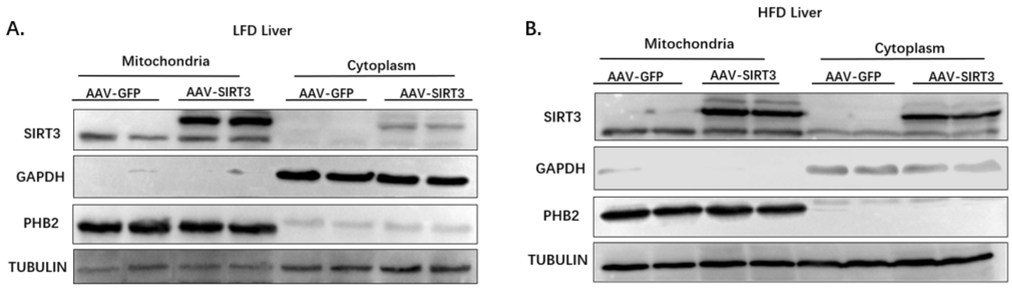

Figure S2. The location of hepatic SIRT3. 5-6 weeks of C57BL/6N male mice were injected AAV-GFP or AAV-SIRT3 via tail vein and fed with LFD and HFD for 12 weeks, respectively. At the 14th weeks of feeding, mice were sacrifized and liver were collected from the mice. A. The location of hepatic SIRT3 in mice under LFD. B. The location of hepatic SIRT3 in mice under HFD. n=4 mice per group.
